# Plant Unsaturated Fatty Acids: Multiple Roles in Stress Response

**DOI:** 10.3389/fpls.2020.562785

**Published:** 2020-09-04

**Authors:** Mei He, Nai-Zheng Ding

**Affiliations:** College of Life Science, Shandong Normal University, Jinan, China

**Keywords:** land plants, unsaturated fatty acids, stress-related roles, genetic engineering, stress tolerance

## Abstract

Land plants are exposed to not only biotic stresses such as pathogen infection and herbivore wounding, but abiotic stresses such as cold, heat, drought, and salt. Elaborate strategies have been developed to avoid or abide the adverse effects, with unsaturated fatty acids (UFAs) emerging as general defenders. In higher plants, the most common UFAs are three 18-carbon species, namely, oleic (18:1), linoleic (18:2), and α-linolenic (18:3) acids. These simple compounds act as ingredients and modulators of cellular membranes in glycerolipids, reserve of carbon and energy in triacylglycerol, stocks of extracellular barrier constituents (e.g., cutin and suberin), precursors of various bioactive molecules (e.g., jasmonates and nitroalkenes), and regulators of stress signaling. Nevertheless, they are also potential inducers of oxidative stress. In this review, we will present an overview of these roles and then shed light on genetic engineering of FA synthetic genes for improving plant/crop stress tolerance.

## Introduction

Land plants are living in a harsh environment that imposes strikingly manifold stresses on their health and productivity. Biotic stressors include viruses, bacteria, fungi, nematodes, and arthropods, among others. The first four pathogens cause various diseases such as leaf spot, stem rust, and root rot, while arthropods are herbivorous or parasitic and may serve as pathogen vector. Abiotic stressors include temperature (low or high), water (deficit or excess), ultraviolet, salt, heavy metals, and so on. High salinity, for example, can induce ionic toxicity, osmotic pressure, oxidative damage, and nutrient deficiency (Zhao et al., 2010; Feng et al., 2014a; Feng et al., 2014b), thereby strongly suppressing the whole lifecycle of most plants (Guo et al., 2012; Guo et al., 2015; Guo et al., 2018). More seriously, in the context of climate change and soil salinization, cold/heat, drought and salt usually arise together, posing an increasingly severe threat to plant subsistence and cultivation worldwide (Yuan et al., 2015; He et al., 2018). Even worse is that biotic stresses may occur simultaneously with them.

**Figure 1:**
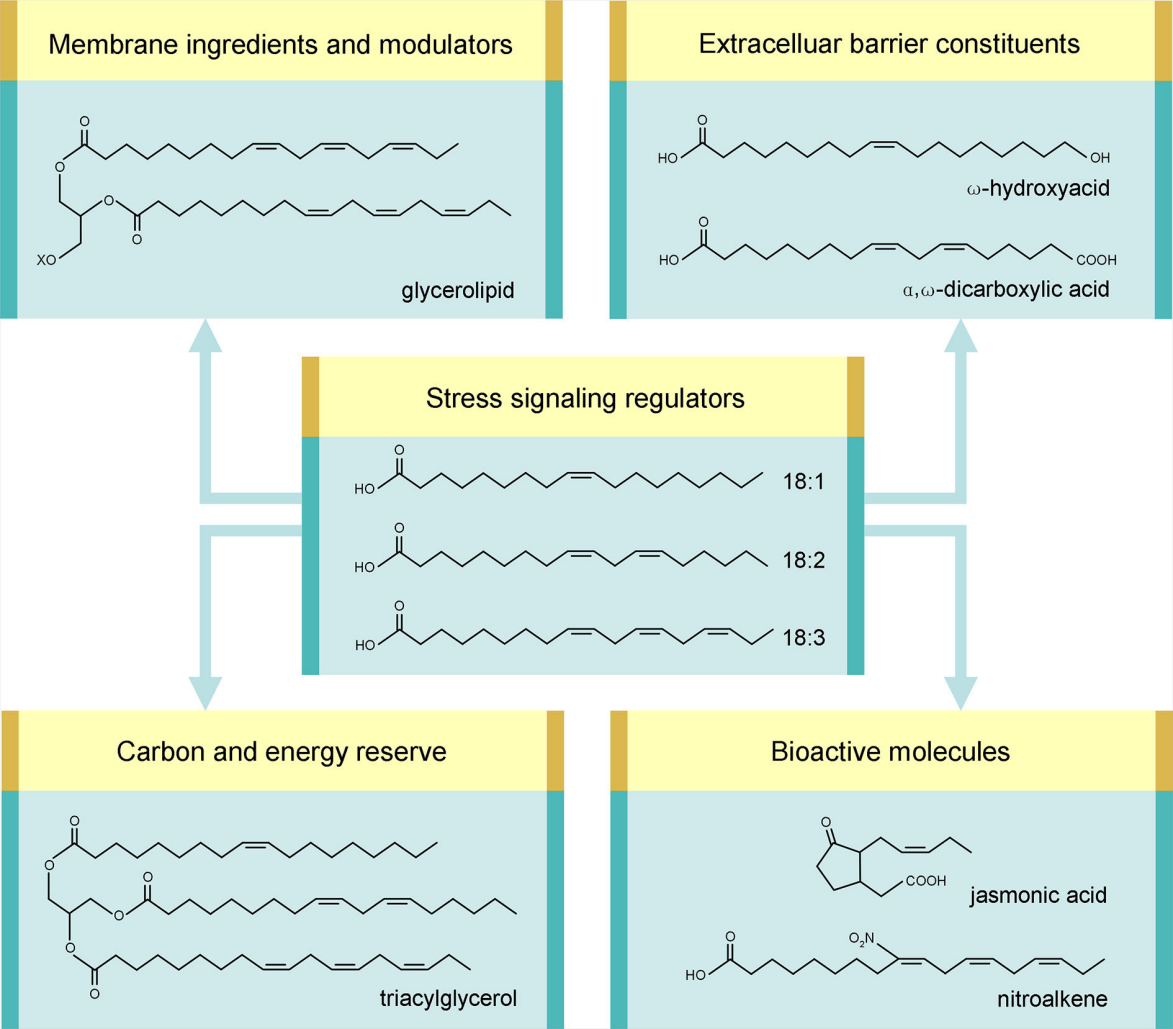
Multiple roles of C18 unsaturated fatty acids in stress defense. Examples of corresponding derivatives are shown. “X” stands for the head group of glycerolipid.

**Figure 2:**
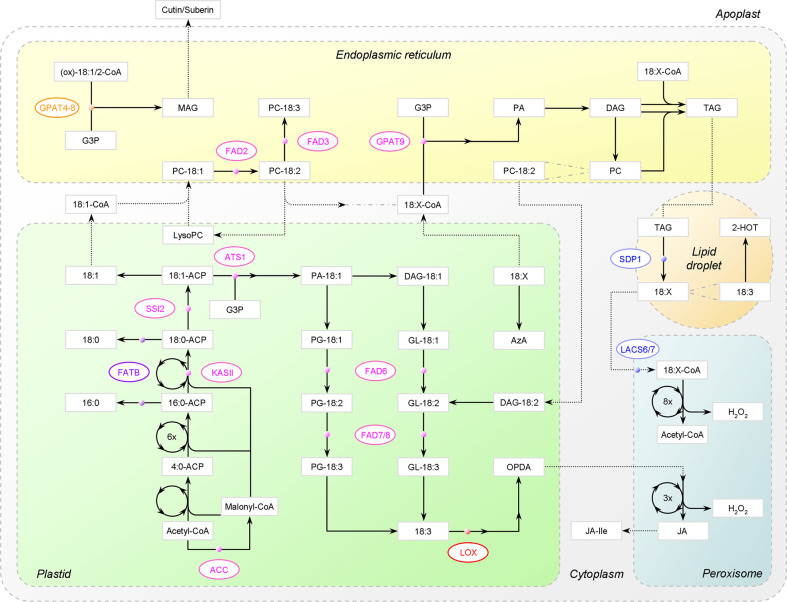
Major metabolic pathways of C18 unsaturated fatty acids (UFAs) in *Arabidopsis*. FA synthesis in plastids and β-oxidation in peroxisomes are depicted as circle. PC-18:2 is used as an example substrate to show the acyl editing way of 18:1 esterification and the eukaryotic way of glycolipid synthesis. Dotted arrow denotes that trafficking is involved. For simplicity, the bypath in JA synthesis, connections between acyl-CoA pools, and connections between 18:X and membrane lipids are not shown. ACC, acetyl-CoA carboxylase; ACP, acyl-carrier protein; KASII, 3-ketoacyl-ACP synthase II; FATB, acyl-ACP thioesterase B; SSI2, stearoyl-ACP desaturase; G3P, glycerol-3-phosphate; GPAT, G3P acyltransferase; ATS1, plastidial GPAT; PA, phosphatidic acid; DAG, diacylglycerol; PG, phosphatidylglycerol; GL, glycolipids, PC, phosphatidylcholine; FAD, fatty acid desaturase; (ox)-18:1/2-CoA, unmodified or oxygenated 18:1/18:2-CoA; MAG, monoacylglycerol; 18:X, C18 UFAs; TAG, triacylglycerol; SDP1, TAG lipase; LACS, long-chain acyl-CoA synthetase; 2-HOT, 2-hydroxy-octadecatrienoic acid; AzA, azelaic acid; LOX, 13-lipoxygenase; OPDA, 12-oxo-phytodienoic acid; JA, jasmonic acid; JA-Ile, jasmonoyl-isoleucine.

**Figure 3:**
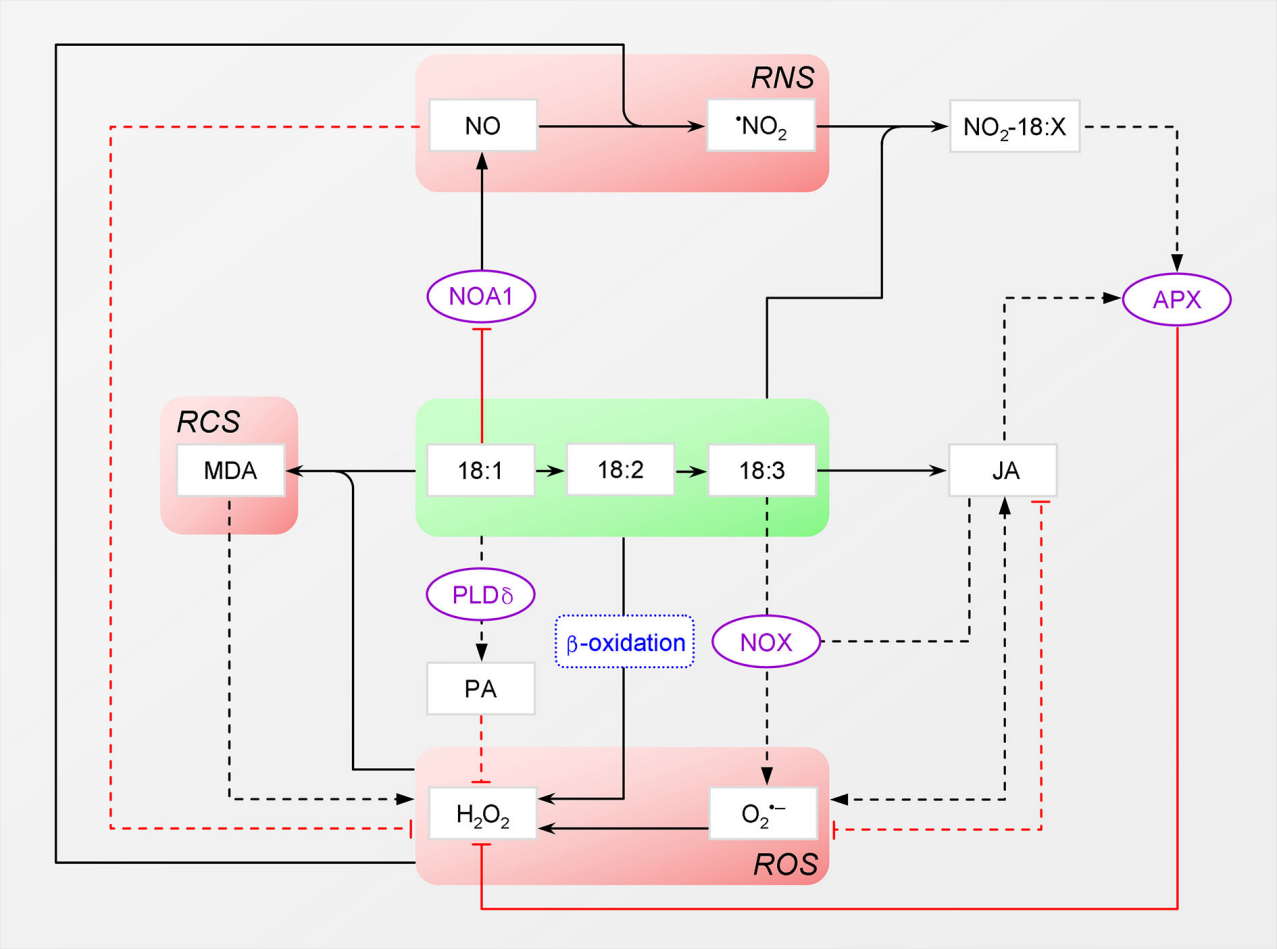
Intertwinement between C18 unsaturated fatty acids and reactive species. Reaction is distinguished from activation and catalysis with different arrowhead. Dashed line denotes indirect effect (e.g., *via* signaling). Please note that the crosstalk between JA and NO is not shown. ROS, reactive oxygen species; RCS, reactive carbonyl species; RNS, reactive nitrogen species; MDA, malondialdehyde; PLDδ, plasma membrane phospholipase D; PA, phosphatidic acid; NOX, NADPH oxidase; JA, jasmonic acid; APX, ascorbate peroxidase; 18:X, C18 unsaturated fatty acids; NOA1, NITRIC OXIDE ASSOCIATED 1.

Being sessile organisms, land plants cannot but face the stresses and hence have evolved elaborate strategies to avoid or abide the adverse effects. Unsaturated fatty acids (UFAs) are coming into the limelight as one of the general defense systems against various biotic and abiotic stresses (He et al., 2018). In higher plants, the most common UFAs are three 18-carbon (C18) species: 18:1 (oleate), 18:2 (linoleate), and 18:3 (α-linolenate), where m:n denotes an FA with m carbon atoms and n *cis*-double bonds (He et al., 2020). Here, we will present an overview of their roles in stress defense as follows: (1) ingredients and modulators of cellular membranes in glycerolipids; (2) reserve of carbon and energy in triacylglycerol (TAG); (3) stocks of extracellular barrier constituents; (4) precursors of various bioactive molecules; (5) regulators of stress signaling (**Figure 1**). In addition, light will be shed on genetic engineering of FA synthetic genes for improving stress tolerance. For better understanding, the metabolism of C18 UFAs (**Figure 2**), as well as the intertwinement between C18 UFAs and reactive species (RS) (**Figure 3**), will be briefly introduced first.

## Metabolism of C18 Unsaturated Fatty Acids

As depicted in **Figure 2**, C18 UFAs stem from acetyl-coenzyme A (CoA) *via de novo* FA synthesis in plastids. 18:0-ACP, the C18 end product attached to acyl-carrier protein, is mostly destined for the unsaturation program conducted by a cascade of FA desaturases (FADs). Once produced, it is rapidly converted to 18:1-ACP by stearoyl-ACP desaturase (SAD). However, not until being esterified into membrane glycerolipids will 18:1(Δ9) be processed to 18:2(Δ9, 12) by ω-6 FADs and further to 18:3(Δ9, 12, 15) by ω-3 FADs, where Δ and ω stand for the carboxylic and methyl ends, respectively [for more, see reviews (Ohlrogge and Browse, 1995; He et al., 2020)].

There are two pathways for membrane glycerolipid biogenesis: the “prokaryotic” one in plastids and the “eukaryotic” one in the endoplasmic reticulum (ER). Both are based on sequential acylation of glycerol-3-phosphate (G3P), with phosphatidic acid (PA) and diacylglycerol (DAG) being the intermediates. The first reaction is catalyzed by G3P acyltransferase (GPAT), rendering it a key enzyme. With the emergence of substrate glycerolipids, C18 polyunsaturated FAs (PUFAs) are then generated by the ω-6 and ω-3 pair of FADs, i.e., plastidial FAD6-FAD7/8 or ER FAD2-FAD3 (Ohlrogge and Browse, 1995; He et al., 2020).

In the eukaryotic pathway, phosphatidylcholine (PC) is the vector for FA desaturation. However, nascent 18:1 exported out of plastids appears to prefer the acyl editing way to be directly incorporated into PC rather than the multistep *de novo* way (He et al., 2020). The exchanged acyl chains (e.g., 18:2) can then enter other metabolic fates, for example, the production of TAG prevailing in seed cells or cutin unlocked in epidermal cells. Of note, unlike PC and TAG that are directly converted from DAG, cutin or suberin is assembled *via* monoacylglycerol (MAG) (Beisson et al., 2012).

Further, C18 UFAs, esterified or released, can be modified into a great variety of bioactive molecules, such as jasmonic acid (JA), azelaic acid (AzA), and 2-hydroxy-octadecatrienoic acid (2-HOT) from oxidation. Nevertheless, when incorporated into TAG, their major fate is to retrograde into acetyl-CoA *via* β-oxidation in peroxisomes. Notably, FAs removed from membrane lipids for turnover will be deposited in TAG first (Fan et al., 2017).

## Intertwinement Between C18 Unsaturated Fatty Acids and Reactive Species

A common stress consequence is the burst of reactive oxygen species (ROS), e.g., superoxide anion (O_2_^•–^) and hydrogen peroxide (H_2_O_2_). ROS can readily attack various biomolecules, with lipids, proteins, carbohydrates, and nucleic acids all being endangered (Saxena et al., 2016). Their excess can thereby bring cells to oxidative catastrophe, including membrane injury, photoinhibition augment, and gene mutation (Sharma et al., 2012; Sui, 2015). Nevertheless, these inevitable toxic byproducts of aerobic metabolism are tactically exploited as signaling molecules to coordinate diverse biological processes, particularly stress defense. They are also actively produced as poisons to retort biotic intruders. Plants have therefore developed sophisticated producing and scavenging systems to adjust ROS levels according to the context. For instance, NADPH oxidase (NOX, or RBOH for respiratory burst oxidase homolog) generates O_2_^•–^, whereas ascorbate peroxidase (APX) eliminates H_2_O_2_ (Gechev et al., 2006; Choudhury et al., 2017).

Due to the presence of double bond, C18 UFAs are vulnerable to ROS. In fact, malondialdehyde (MDA), a product of their peroxidation, is widely used as an indicator of oxidative stress (Guo et al., 2012). Notably, MDA is a latent reactive carbonyl species (RCS) capable of launching a new round of attack under acidic conditions. By forming covalent adducts termed advanced lipoxidation end-products (ALEs), this RCS can cause protein malfunction and consequent ROS proliferation (Farmer and Davoine, 2007; Deng et al., 2010). C18 UFAs are hence turned from victims into accomplices. Moreover, FA β-oxidation is a source of H_2_O_2_, while 18:3 is a likely stimulator of NOX, which will be discussed later on.

On the contrary, the chemical nature also renders C18 UFAs intrinsic antioxidants; that is, they can directly react with and thus consume ROS. And their oxidation gives rise to various oxylipins, as represented by the stress hormone JA, which, in turn, modulates ROS levels and signaling (see below). Interestingly, 18:3, though less effective than 18:1, can also stimulate the plasma membrane (PM) phospholipase D (PLDδ) that generates PA to attenuate H_2_O_2_-induced cell death in *Arabidopsis* (Wang and Wang, 2001; Zhang et al., 2003). Further, the participation of nitric oxide (NO) (see below), the primary reactive nitrogen species (RNS), adds one more layer of complexity to the intertwinement between C18 UFAs and RS (**Figure 3**).

## Multiple Roles of C18 Unsaturated Fatty Acids in Stress Defense

*In planta*, C18 UFAs are utilized as raw material to produce numerous aliphatic compounds, including membrane glycerolipids, TAG, cutin/suberin, jasmonates, and nitroalkenes (NO_2_-FAs). It is remarkable that all these products, as well as C18 UFAs themselves, take part in plant defense against various biotic and abiotic stresses. C18 UFAs are therefore implicated, directly and indirectly, in stress defense *via* multiple mechanisms (examples listed in **Table 1**), which will be discussed hereinafter.

**Table 1:** Mechanisms for C18 unsaturated fatty acids in stress defense.

Related molecule	Mechanism
Membrane glycerolipids	Maintaining proper membrane fluidity required for multiple membrane-dependent processes, e.g., Ca^2+^ signaling
	Modulating directly activities of membrane-bound proteins, e.g., plasma membrane (PM) H^+^-ATPase
	Mitigating stress-enhanced photoinhibition on photosystem II
	Signaling to regulate stress defense, e.g., phosphatidic acid (PA) can antagonize H_2_O_2_ in inducing cell death
Triacylglycerol	Supplying carbon and energy for stress survival or recovery
Cutin/Suberin	Constructing physically and chemically resistant barrier
Jasmonates	Signaling to organize local and/or systemic defense
Nitroalkenes	Signaling to motivate heat shock proteins and antioxidant enzymes
118:1/2/3	Stimulating PM phospholipase D (PLDδ) and thus PA production
18:1	Suppressing NITRIC OXIDE ASSOCIATED 1 (NOA1) and thus NO production
18:3	Stimulating PM NADPH oxidase (NOX) and thus O_2_^•–^ production

### Ingredients and Modulators of Cellular Membranes

A main building block of biomembranes is glycerolipid composed of a glycerol core attached with a polar “head” group and two nonpolar “tails” derived primarily from C16/C18 FAs (**Figure 1**). The acyl chains inside the bilayer have a profound impact on membrane properties. Particularly, their unsaturation degree is a major factor shaping membrane fluidity. A higher unsaturation degree can lead to a more fluid state because a *cis*-double bond creates a kink as steric hindrance in intermolecular package (He et al., 2018). Of the membrane-constituting UFAs, C18 species are prominent modulators of unsaturation degree, since *trans*-16:1 has a high melting point and 16:3 is only present in several plants (Ohlrogge and Browse, 1995).

Biomembranes serve as not only structural barrier for cells and intracellular organelles, but functional platform for multiple cellular processes, including substance exchange, signal transduction, and many metabolic reactions. A notable example is that the signaling of Ca^2+^, a versatile second messenger active in plant response to virtually every stress, is based on membrane isolation and transportation. Ca^2+^ signaling is ignited by sharp influx of the cation into the cytosol through its channels, including the PM cyclic nucleotide-gated channels (CNGCs) and the tonoplast TWO PORE CHANNEL 1 (TPC1) (Lu et al., 2016; Zheng et al., 2017), and is quickly quenched by efflux through Ca^2+^-ATPases and Ca^2+^/H^+^ exchangers (Han et al., 2011; Han et al., 2012).

Some other ion transporters, including the K^+^ rectifier ARABIDOPSIS K^+^ TRANSPORTER 1 (AKT1) (Ren et al., 2013) and the Na^+^/H^+^ antiporter SALT OVERLY SENSITIVE 1 (SOS1) (Deng et al., 2016; Kong et al., 2016), maintain a properly high K^+^/Na^+^ ratio in the cytoplasm, which is critical for salt tolerance (Chen et al., 2005; Feng et al., 2015). These secondary transporters are energized by virtue of the electrochemical gradient created by transmembrane proton pumps, e.g., PM H^+^-ATPase, vacuolar H^+^-ATPase (V-ATPase), and vacuolar H^+^-translocating inorganic pyrophosphatase (V-PPase) (Chen et al., 2010; Yang et al., 2010; Yuan et al., 2016). Notably, the PM H^+^-ATPase is a key site responsive to not only salt, but other stresses like cold and heavy metals that also involve active transport across the PM (Martz et al., 2006; Shi et al., 2008; Janicka-Russak et al., 2012).

Besides bulk lipids that can alter membrane fluidity, the conformation and function of a membrane-bound protein may be affected by annular (selectively enriched) or non-annular (tightly bound) lipids (Contreras et al., 2011). As in the case of the PM H^+^-ATPase, its catalytic activity has an absolute requirement for phospholipid, albeit the mechanisms underlying such regulation are still uncertain [see reviews (Kasamo, 2003; Morales-Cedillo et al., 2015)]. When the stimulatory effect of PC was tested *in vitro*, the ATPase activity decreased with increased FA chain length or unsaturation degree, and maximum activation was achieved with the combination of 14:0 and 18:1 (Kasamo, 1990). Free FAs and lysoPC, the hydrolytic products of PC, could also activate the enzyme, with 18:3 being the most effective C18 species (Palmgren et al., 1988). *In vivo*, however, the ATPase activity appears to have a correlation with double bond index (DBI) rather than individual C18 UFAs (Martz et al., 2006; Shi et al., 2008).

Membrane fluidity is susceptible to multiple stresses, especially extreme temperatures. However, both cold-induced rigidification and heat-induced fluidization are detrimental to membrane function, resulting in protein deactivation, electrolyte leakage, and even cytoskeleton destabilization (Sangwan et al., 2002; Liu et al., 2013; He et al., 2018). In fact, being a thermodynamic property, membrane fluidity may serve as a sensor in temperature signaling (Ruelland and Zachowski, 2011). Interestingly, the effects of cold and heat can be mimicked at 25°C by dimethylsulfoxide (DMSO) and benzyl alcohol (BA), respectively. In alfalfa (*Medicago sativa*), the two chemicals induced opposite changes in membrane fluidity coupled with cytoskeleton disassembly, which triggered Ca^2+^ signaling to activate two distinct mitogen-activated protein kinase (MAPK) cascades, leading to corresponding stress response (Sangwan et al., 2002).

Plants are poikilothermic organisms, highlighting the especial importance of membrane remodeling, not to mention the pressure from climate change. Adjusting the unsaturation degree of FA tails is an efficient strategy favored by plants in offsetting thermal perturbations to keep membrane fluidity in the optimal range, as manifested by the temperature sensitivity of *FAD* mutants [see review (Wallis and Browse, 2002)] or transformants (**Table 2**). Particularly, the unsaturation degree of chloroplast phosphatidylglycerol (PG) is a major factor affecting cold tolerance [see reviews (Nishida and Murata, 1996; Iba, 2002)].

**Table 2:** Altered stress tolerance upon genetic manipulation of fatty acid synthetic genes.

Enzyme	Gene^a^	Genetic manipulation^b^	Stress tolerance^c^	Reference
Stearoyl-ACP desaturase	*SSI2/FAB2*	OE in *Brassica napus* from *Sapium sebiferum*	↑freezing	(Peng et al., 2018)
ω-6 Fatty acid desaturase	*FAD2*	OE in *Oryza sativa*	↑cold	(Shi et al., 2012)
ω-3 Fatty acid desaturase	*FAD3*	OE in *Nicotiana tabacum* from *B. napus*	↑drought	(Zhang et al., 2005)
		OE in *Lycopersicon esculentum*	↑cold	(Yu et al., 2009)
		OE in *L. esculentum*	↑salt	(Wang et al., 2014)
		OE in *N. tabacum* from *Chorispora bungeana*	↑cold, drought and salt	(Shi et al., 2018)
	*FAD7*	OE in *N. tabacum* from *Arabidopsis thaliana*	↑cold	(Kodama et al., 1994)
		CS in *N. tabacum* from *A. thaliana*	↑heat	(Murakami et al., 2000)
		AS in *N. tabacum* from *A. thaliana*	↓drought and salt	(Im et al., 2002)
	*FAD8*	OE in *N. tabacum* from *A. thaliana*	↑drought	(Zhang et al., 2005)
Glycerol-3-phosphate acyltransferase	*ATS1/ACT1*	OE in *N. tabacum* from *A. thaliana*	↑cold	(Murata et al., 1992)
		AS in *L. esculentum*	↑heat	(Sui et al., 2007)
		OE in *A. thaliana* from *Suaeda salsa*	↑salt	(Sui et al., 2017)
		OE in *A. thaliana* from *Ammopiptanthus mongolicus*	↑cold, freezing and oxidative	(Xue et al., 2019)

a Arabidopsis gene name.

b OE, overexpression; CS, co-suppression; AS, antisense suppression.

c ”↑”, enhanced; “↓”, reduced.

Being the sole phospholipid species present in thylakoid membranes, PG is an indispensable constituent of the membrane-bound photosynthetic apparatus, e.g., photosystem II (PSII) (Wada and Murata, 2007). PSII is subject to photoinhibition, wherein the D1 protein of the reaction center is caught in an endless loop of photodamage and repair (Takahashi and Murata, 2008; Liu et al., 2016). PG desaturation is capable of protecting PSII from cold-enhanced photoinhibition (Moon et al., 1995). This may apply to other stresses that can potentiate the process as well (Takahashi and Murata, 2008). Under saline conditions, alleviated PSII photoinhibition has been observed to be accompanied with elevated UFA content in membrane lipids (Sui et al., 2010; Sui and Han, 2014; Liu et al., 2017). Indeed, specifically raising the unsaturation level of PG could accelerate the repair of D1 (Sun et al., 2010).

However, it should be pointed out that the relationship between membrane (particularly PM) lipid unsaturation and salt tolerance are elusive (examples listed in **Table 3**). For salt-sensitive species, it is plausible that increase and decrease in the unsaturation degree reflect defense and damage, respectively, depending on their sensitivity to certain salinity. This is supported by the behavior of peanut (*Arachis hypogaea*) that is moderately tolerant to salt — the DBI of total leaf lipids went up at 150 mM NaCl, but fell down at higher concentrations (Liu et al., 2017; Sui et al., 2018). Notably, choline (Salama and Mansour, 2015) and silicon (Liang et al., 2006) were able to reverse the reductions in PM unsaturation of wheat (*Triticum sativum*) and PM fluidity of barley (*Hordeum vulgare*), respectively, which contributed to improved salt tolerance.

**Table 3:** Changes in membrane lipid unsaturation under salt stress.

Species		Tissue	Tolerance	Membrane	Lipid unsaturation^a^			Reference
					no/lower NaCl	highest NaCl	change	
single	*Brassica oleracea*	root	tolerant	plasma	137*	171*	↑	(López-Pérez et al., 2009)
	*Carthamus tinctorius*	root	tolerant	total	85.21*	126.37*	↑	(Harrathi et al., 2012)
	*Suaeda salsa*	leaf	halophyte	total	126.7*	144.7*	↑	(Sui et al., 2010)
	*Triticum aestivum*	root	sensitive	plasma	1.9	1.5	↓	(Mansour et al., 1994)
	*Glycine max*	root	sensitive	plasma	1.04	0.79	↓	(Surjus and Durand, 1996)
pair	*Hordeum maritimum*	root	tolerant	total	1.30	1.26	→	(Chalbi et al., 2013)
	*Hordeum vulgare*		sensitive		0.46	1.02	↑	
	*Thellungiella halophila*	leaf	halophyte	total	73.59*	124.79*	↑	(Sui and Han, 2014)
	*Arabidopsis thaliana*		sensitive		104.03*	51.06*	↓	
	*Buchloe dactyloides*	root	tolerant	plasma	23*	123*	↑	(Lin and Wu, 1996)
			sensitive		–	66*	↑	
	*Lycopersicon esculentum*	callus	tolerant	plasma^b^	45.5*	32.4*	↓	(Kerkeb et al., 2001)
			sensitive		46.1*	n/a	n/a	
	*Zea mays*	root	tolerant	plasma	0.62	0.24	↓	(Salama et al., 2007)
			sensitive		0.54	0.46	↓	
	*Brassica napus*	root	tolerant	plasma	0.60, 0.65	0.28, 0.22	↓	(Zamani et al., 2010)
			sensitive		0.54-0.55	0.44-0.46	↓	

a Unsaturation to saturation ratio if not labeled; ^b^phosphatidylcholine unsaturation; “*”, double bond index; “–”, Not detected; “↑”, increase; “→”, little change; “↓”, decrease.

b Phosphatidylcholine unsaturation.

Surprisingly, increasing, maintaining and decreasing the unsaturation degree are instrumental for different salt-tolerant species. Of species like maize (*Zea mays*), the tolerant varieties exhibited a greater decline than did the sensitive ones upon NaCl treatment (**Table 3**). Such change was assumed to reduce PM fluidity and thus the permeability and import of Na^+^ and Cl^−^, thereby alleviating salt stress. Another possibility is that, in these species, while other factors (e.g., sterol composition) are more involved in modulating PM properties, UFA content is lowered to mitigate PM susceptibility to salt-induced oxidative damage [see review (Guo et al., 2019)]. Nevertheless, gain- or loss-of-function of FADs has demonstrated the positive effect of FA unsaturation on salt tolerance [**Table 2** and (Guo et al., 2019)]. Of note, even though tomato (*Lycopersicon esculentum* or *Solanum lycopersicum*) fell into the decreasing group (**Table 3**), *FAD3* overexpression did enhance the ability of early seedlings to resist high salinity (**Table 2**).

### Reserve of Carbon and Energy

FAs are incorporated into TAG (**Figure 1**) and packaged in lipid droplets (or oil bodies) as carbon and energy reserve. In seed cells, TAG is accumulated for fueling seedling establishment, which is fundamental to plant revival after escaping biotic and abiotic stresses *via* seed dormancy. When conditions become suitable for germination, FAs are channeled to metabolic breakdown *via* the β-oxidation spiral operating in glyoxysomes, the specialized peroxisomes (**Figure 2**). Acetyl-CoA is thereby regenerated, which is a core metabolite for energy production *via* mitochondrial respiration and for carbohydrate anabolism *via* the glyoxylate cycle and gluconeogenesis [see reviews (Graham, 2008; Xu and Shanklin, 2016)]. Noteworthily, acetyl-CoA is also the donor for histone acetylation, conferring peroxisomal FA β-oxidation a regulatory role in nuclear epigenetic modification, which may affect diverse cellular processes (Wang et al., 2019).

In *Arabidopsis*, the mobilization process has been characterized, though not fully understood. Firstly, FAs are liberated by a series of lipases, including the TAG lipase SUGAR-DEPENDENT1 (SDP1) (Eastmond, 2006) and the MAG lipase MAGL8 (Kim et al., 2016). A likely route for peroxisome import is that FAs are transferred as CoA esters across the envelope by the peroxisomal ATP-binding cassette (ABC) transporter PXA1 (also ABCD1, CTS or PED3); however, CoA will be lost due to the intrinsic acyl-CoA thioesterase activity of PXA1 (De Marcos Lousa et al., 2013). LACS6 and -7, two long-chain acyl-CoA synthetases, are then required to re-activate FAs. Subsequently, stepwise chain truncation by β-oxidation gives rise to acetyl-CoA per round [for detail, see review (Graham, 2008)].

Notably, TAG is more than an energy-dense storage form. It appears to be a key intermediate in peroxisomal degradation of membrane-constituting FAs. In other words, to enter β-oxidation, FAs removed from membrane lipids will be deposited in TAG first (Fan et al., 2017). In vegetative tissues, despite the high synthetic capacity, TAG yield is constrained due to biased partitioning toward membrane glycerolipid production and rapid SDP1-driven TAG turnover (Xu and Shanklin, 2016). Upon carbon depletion that can be induced by extended darkness, chloroplast lipids will be mobilized as substitutes for respiration. However, when *Arabidopsis sdp1* mutants were under this situation, 16:3 unique to the plastid-intrinsic monogalactosyldiacylglycerol (MGDG) accumulated in TAG. The even distribution of 16:3 across the three positions of TAG suggested that 16:3 was first hydrolyzed and then exported to the ER for *de novo* TAG synthesis (Fan et al., 2017).

Indeed, PLASTID LIPASE 1 (PLIP1), a thylakoid-associated phospholipase A_1_, has been identified to drive the turnover of PG with *sn-2 trans*-16:1 by cleaving *sn-1* PUFAs, primarily 18:3. In seed cells, the liberated 18:3 is eventually incorporated into TAG, with PC serving as either DAG or acyl donor (Wang et al., 2017). Of note, free FAs, as well as PA and DAG, are cytotoxic. TAG accumulation hence plays a pivotal role in sequestering them into lipid droplets, thereby buffering lipid homeostasis and protecting cells against lipotoxic death due to FA overload (Fan et al., 2017), which can be a consequence of robust membrane remodeling in stress response. Under heat stress, for instance, HEAT INDUCIBLE LIPASE 1 (HIL1) removes 18:3 from MGDG to decrease membrane fluidity, resulting in increased level of 18:3-containing TAG (Higashi et al., 2018).

Nevertheless, FA β-oxidation is a double-edged sword for plants in that a byproduct is H_2_O_2_ (**Figure 2**). For H_2_O_2_ scavenging, peroxisomes deploy double insurance — catalase (CAT) present in the matrix to protect themselves plus the APX system associated with the membrane to prevent leakage into the cytoplasm (Graham, 2008). If H_2_O_2_ generation exceeds detoxification, it is natural that FA β-oxidation will be turned into an inducer of oxidative stress and ultimate cell death, as happened under extended darkness, rendering TAG accumulation another protecting role (Fan et al., 2017).

TAG mobilization may therefore be subjected to opposite regulation, according to whether the supply of carbon skeletons and energy or the avoidance of excess ROS is given priority. To counteract salt stress, melatonin, a phytohormone to be licensed, promotes TAG breakdown in parallel with ROS scavenging, so that enough ATP is provided to sustain the enhanced PM H^+^-ATPase activity, thereby improving K^+^/Na^+^ homeostasis in sweet potato (*Ipomoea batatas*) (Yu et al., 2018). In contrast, *Arabidopsis pxa1* mutants, in which FA import into peroxisomes is blocked, exhibit reduced ROS accumulation and enhanced salt tolerance (Yu et al., 2019).

### Stocks of Extracellular Barrier Constituents

To cope with and adapt to the harsh environment during terrestrial colonization, land plants have developed the frontier tissue with physically and chemically resistant barriers. Basically, the whole plant body is enveloped by a monolayer epidermis. In organs undergoing secondary growth, e.g., roots and stems of woody species, a multilayer periderm evolved instead. Epidermis and periderm are both equipped with FA-derived biopolyesters (cutin and suberin, respectively) to specialize the cell walls. Their principal roles are to participate in restricting non-stomatal fluxes of water, solutes and gases, as well as defending abiotic and biotic stresses, particularly desiccation and wounding. Unlike cutin that is specific to epidermis, suberin is also present in some internal tissues like root endodermis and appears at abscission zones for sealing [for review, see (Pollard et al., 2008; Beisson et al., 2012; Graça, 2015; Fich et al., 2016)].

Cutin is made mostly of C16 (16:0) and C18 (18:0-18:2) FA derivatives generated from ω-terminal and/or mid-chain oxidation, including ω-hydroxyacids, α, ω-dicarboxylic acids (DCAs) (**Figure 1**), epoxyacids, and polyhydroxyacids (Beisson et al., 2012). Based on the ester bond formed between carboxyl (–COOH) and hydroxyl (–OH), these monomers are linked directly to each other or, to a much lesser extent, *via* a bridging molecule such as glycerol or ferulic acid (Pollard et al., 2008; Deng et al., 2015). The resultant macromolecule serves as the framework to be embedded with and coated by cuticular waxes, a complex mixture consisting chiefly of saturated very long-chain FA (VLCFA) derivatives. A translucent hydrophobic film termed cuticle is thereby constructed on the polysaccharide matrices of primary cell walls to seal the aerial surface of epidermis (Fich et al., 2016; He et al., 2018).

Different from cutin, suberin utilizes saturated VLCFAs in addition to C16/18 FAs as monomer precursors to be oxygenated, and usually contains a higher amount of DCAs, primary alcohols, glycerol, and phenolics (Beisson et al., 2012). Particularly, it is covalently combined with lignin-like polyaromatics, in which phenolics are interlinked by C–C or ether (C–O–C) bonds that are more hydrolysis-resistant. A typical polylamellate secondary wall known as suberin lamellae is thereby built beneath the primary wall of a suberized cell (Pollard et al., 2008; Graça, 2015). Of note, herein, suberin denotes the aliphatic polyester only rather than a macromolecule containing polyaliphatic and polyaromatic domains, as was proposed by Graça, (2015).

The monomer composition of cutin or suberin can vary greatly with plant species, organ, and developmental stage. In *Arabidopsis*, for example, the cutin of leaves and stems has an unusual high content of 18:2-derived DCA (Bonaventure et al., 2004), whereas that of flowers is mainly assembled from 16:0-derived dihydroxyacid (Li-Beisson et al., 2009). Moreover, it is worth noting that unmodified FAs may also appear in cutin or suberin, acting like primary alcohols as dead ends in the polyester (Pollard et al., 2008; Graça, 2015).

The major themes of monomer biosynthesis common to cutin and suberin have been delineated in *Arabidopsis*. For polymerization, the aliphatic monomers are in the form of *sn*-2 MAG. The intermediates are generated from free FAs *via* acyl activation, oxidation, and esterification to glycerol, albeit the order of this pathway has not been fully determined (Pollard et al., 2008; Fich et al., 2016). LACS members are required in that the ER-localized GPAT members utilize acyl-CoAs as substrates. Of acyl oxidation reactions, hydroxylation is better characterized, with members of the CYP86 and CYP77 subfamilies of cytochrome P450 monooxygenases being ω-terminal and mid-chain hydroxylases, respectively (Beisson et al., 2012).

It appears that different isozymes are used for different context, as exemplified by GPATs — GPAT4/8 in leaves and stems vs. GPAT6 in flowers for cutin, while GPAT5 in roots and seeds vs. GPAT7 likely in wounded tissues for suberin (Yang et al., 2012). Notably, GPAT substrate specificity is a major factor accounting for the difference in monomer composition between cutin and suberin. GPAT4, -6, and -8 are strongly biased toward C16/18 ω-oxidized acyl-CoAs, whereas GPAT5 can accept a broader chain length range of ω-oxidized and unmodified acyl-CoAs (Yang et al., 2012). Interestingly, ectopic expression of GPAT5 together with CYP86A1 or -B1 resulted in the appearance of C20-24 suberin monomers in stem cutin (Li et al., 2007; Molina et al., 2009).

To assemble the apoplastic polyesters, the materials need to be exported from the ER to and then across the PM. For cutin, additional transit through the cell wall onto the outer surface follows. ABC transporter members are engaged in channeling the PM for cutin/suberin (Beisson et al., 2012). Particularly, the half-transporter ABCG11 is shared by them (Panikashvili et al., 2010). Recently, LTPG15, a PM glycosylphosphatidylinositol (GPI)-anchored lipid transfer protein, was identified to participate in suberin monomer export in seed coat (Lee and Suh, 2018). Like transport means, both polymerization mechanism and polymer architecture remain largely elusive. The formation of cutin is beginning to be understood with the discovery of CUTIN SYNTHASE 1 (CUS1), a cuticle-localized member of the GDSL lipase/hydrolase superfamily (Girard et al., 2012; Yeats et al., 2012). Nevertheless, given that the *CUS1* null mutant of tomato is not fully deprived of fruit cutin, non-catalytic ways cannot be ruled out yet (Yeats et al., 2012; Fich et al., 2016).

In addition, there are other protective biopolymers composed of aliphatics and aromatics, including cutan and sporopollenin. Cutan is present in a few plants as an additional cuticle fraction (Gupta et al., 2006), with 18:2 appearing to be preferred over 16:0 among the FA precursors (Villena et al., 1999). Sporopollenin is the basic component of the exine that covers a spore/pollen grain, with 18:1 being one of the aliphatic sources [see review (Ariizumi and Toriyama, 2011)]. Being refractory to degradation, however, the two macromolecules are so far less characterized.

### Precursors of Various Bioactive Molecules

As aforementioned, C18 UFAs, esterified or released, can be modified into a great variety of bioactive molecules. Besides JA, AzA and 2-HOT from oxidation, examples include NO_2_-FAs and *N*-acylethanolamines (NAEs) from nitridation. Like JA and NO_2_-FAs (**Figure 1**) addressed below, NAEs, though less understood, are signaling molecules implicated in diverse biological processes [see review (Hou et al., 2016)]. By contrast, AzA and 2-HOT are more specific for defending biotic stresses. While 2-HOT (from 18:3 of TAG) is an antifungal compound [see review (Shimada et al., 2018)], AzA (from C18 UFAs of galactolipids) is an inducer of systemic acquired resistance (SAR) against secondary pathogen infection by promoting G3P biosynthesis [see review (Lim et al., 2017)].

#### Jasmonates

JA and its derivatives, as well as its precursor 12-oxo-phytodienoic acid (OPDA), compose the jasmonate family of phytohormones. JA is derived from 18:3 or 16:3 of plastidial glycerolipids *via* clear enzymatic pathways, with 13-lipoxygenase (LOX) catalyzing the first oxygenation step. Recently, it was observed that OPDA can directly enter β-oxidation to create 4,5-didehydro-JA first, thus bypassing the canonical octadecanoid pathway sketched in **Figure 2** [for more, see reviews (Wasternack and Strnad, 2018; Ruan et al., 2019)]. Another notable update is that PLIP2 and PLIP3, two putative paralogs of the aforecited PLIP1, are capable of triggering JA synthesis in response to abscisic acid (ABA) by releasing 18:3 from PG and MGDG, respectively (Wang et al., 2018). A new node is then added to the crosstalk between the two prominent stress hormones, accounting for the induction effect of ABA on JA.

Well-known in organizing wounding response, JA is active in combating various other stresses, including drought (Liu et al., 2018; Luo et al., 2019) and salt (Yang et al., 2017; Yuan et al., 2019). Particularly, it is of economic interest that methyl JA (MeJA) is capable of alleviating chilling injury to fruits and vegetables, and thus can be applied to maintain their post-harvest quality (González-Aguilar et al., 2000; Ding et al., 2002). Moreover, it is noteworthy that MeJA can induce epigenetic changes of defense-related genes, thereby priming plants against future biotic and abiotic stresses (Laura et al., 2018). In stress response, JA extensively interplays with other signaling molecules involved, as a synergist or antagonist. Here, the focus is on the intricate crosstalk between JA and H_2_O_2_.

Briefly, in canonical JA signaling, the primary ligand is the conjugate jasmonoyl-isoleucine (JA-Ile). The receptor, however, requires two components. One is CORONATINE INSENSITIVE 1 (COI1), a subunit of the Skp1/Cullin/F-box (SCF) E3 ubiquitin ligase; the other is the jasmonate ZIM domain (JAZ) family of transcription factors (TFs) that, through direct interaction, can repress various other TFs, typically the key activator MYC2. Upon elicitation, JA-Ile mediates the binding of COI1 to JAZs, leading to their ubiquitination and consequent proteasomal degradation. MYC2 is then released to turn on its target genes, thereby launching robust JA response [for more, see reviews (Wasternack and Hause, 2013; Ali and Baek, 2020)]. More specifically, in cold tolerance, TFs belonging to the C-REPEAT BINDING FACTOR/DEHYDRATION RESPONSIVE ELEMENT BINDING FACTOR 1 (CBF/DREB1) family play a central role *via* activating downstream targets such as the cold-regulated (*COR*) genes (He et al., 2016; Wang et al., 2016). These TFs themselves are subject to transactivation by the inducer of CBF expression (ICE) TFs. However, ICEs are repressed by JAZs, rendering JA a positive regulator of this anti-cold cascade (Hu et al., 2013).

As already mentioned, H_2_O_2_ possesses signaling property. In fact, plant cells employ diverse enzymes to actively produce H_2_O_2_ to launch local and/or systemic defense against biotic and abiotic stresses. The major one is the PM-localized NOX that generates O_2_^•–^. This radical is soon converted to H_2_O_2_ spontaneously or by superoxide dismutase (SOD). A core component of H_2_O_2_ signaling is the MAPK pathway composed of three kinases: MAPK kinase kinase (MAPKKK), MAPK kinase (MAPKK/MKK), and MAPK. Of note, MAPK cascades can differentially decipher H_2_O_2_ signals. MPK3 and MPK6, two MAPKs, are more characterized as stress-related transducers. Moreover, reversible thiol oxidation of target proteins may offer a direct and important way for H_2_O_2_ signal transduction [for more, see reviews (Jalmi and Sinha, 2015; Liu and He, 2017; He et al., 2018)].

JA can affect H_2_O_2_ producing and/or scavenging processes, depending on the context. When tomato leaves were mechanically wounded, JA was rapidly synthesized and then increased NOX activity to accumulate H_2_O_2_ as its second messenger to switch on an array of defensive genes, e.g., those encoding for proteinase inhibitors (Orozco-Cárdenas et al., 2001). Actually, JA biosynthesis *per se* is a source of H_2_O_2_ (**Figure 2**), in which three or two rounds of β-oxidation are involved (Ruan et al., 2019). In contrast, when *Arabidopsis* was infected by the bacterium *Pseudomonas syringae* DC3000, MeJA induced *NATA1* expression to reduce H_2_O_2_ generation from polyamine oxidation. The encoded N-ACETYLTRANSFERASE ACTIVITY 1 acetylated putrescine, the precursor of spermidine and spermine, thereby lessening their amount for oxidation (Lou et al., 2016). Moreover, as demonstrated by mounting evidence, JA can modulate the expression and/or activities of various antioxidant enzymes, including CAT, APX and SOD (Hanaka et al., 2016; Ali and Baek, 2020).

Inversely, H_2_O_2_ can up-regulate JA biosynthetic genes (e.g., *LOX*), as has been observed in tobacco (*Nicotiana tabacum*) (Vandenabeele et al., 2003). This effect is likely mediated by MPK3, given that wound-induced protein kinase (WIPK), a tobacco MPK3, is required for JA production (Seo et al., 1999), and that introducing a peanut ortholog into tobacco resulted in higher transcription of *LOX* (Kumar et al., 2009). Notably, in tomato (Kandoth et al., 2007) and rice (*Oryza sativa*) (Wang et al., 2013), MPK3 targets *LOX* as well. Interestingly, in *Arabidopsis*, JA can activate the MKK3-MPK6 cascade to repress *MYC2* expression, thereby fine-tuning its own signaling (Takahashi et al., 2007). Since MAPK cascades can shape H_2_O_2_ signaling *via* modulating, for example, NOX activity (Jalmi and Sinha, 2015), it is possible that they also participate in mediating JA regulation on H_2_O_2_. Further, if JA-induced MPK6 activation is independent of H_2_O_2_ (e.g., *via* Ca^2+^), then MPK6 renders a converging node for the two signaling pathways. Similarly, Ca^2+^ may also participate in the crosstalk between JA and H_2_O_2_ as a mediator and integrator.

#### Nitroalkenes

Nitro-FAs, primarily NO_2_-FAs, are newly recognized signaling molecules common to plants and animals. These species are created by the spontaneous reaction between UFA and nitrogen dioxide (·NO_2_) (**Figure 3**), an RNS derived from NO, nitrite (NO_2_ˉ), or peroxynitrite (ONOOˉ). Endogenous NO_2_-FAs can be free, lipid-esterified or protein-adducted [see reviews (Schopfer et al., 2011; Mata-Pérez et al., 2018)]. In the plant kingdom, the first to be discovered were NO_2_-18:1-Cys adducts in fresh olives and conjugated NO_2_-18:2 in extra virgin olive oil (Fazzari et al., 2014). Subsequently, NO_2_-18:3 was identified in several model species, including *Arabidopsis*, rice and pea (*Pisum sativum*) (Mata-Pérez et al., 2016; Mata-Pérez et al., 2017).

Based on their chemical nature, there are two ways known for NO_2_-FAs to exert signaling character in animals. One is to be NO donors that can release the gasotransmitter into aqueous microenvironments, thereby triggering NO signaling to regulate diverse biological processes (Mata-Pérez et al., 2018). Notably, NO is capable of protecting plants against a wide spectrum of stresses, not least oxidative stress (Kong et al., 2016; He et al., 2018). The other is to be protein modulators that can induce post-translational nitroalkylation. Like aldehyde, the electron-withdrawing NO_2_ group turns the UFA moiety into an electrophile, enabling it to attack nucleophiles, including Cys, His and Lys residues, *via* a reversible reaction termed “Michael addition”. The modification activates or represses target proteins, including signal transducers, TFs and metabolic enzymes, leading to substantial reprogramming (Schopfer et al., 2011).

In *Arabidopsis*, NO_2_-18:3 has been found to be significantly elevated under various abiotic stresses, including cold, salt, cadmium, and mechanical wounding. Transcriptomic analysis revealed that it can induce the expression of heat shock proteins (HSPs) and an APX isoform (Mata-Pérez et al., 2016). HSPs are representative molecular chaperones serving as a universal salvation system against not only heat but all other adverse conditions that can cause protein damage. They can prevent denatured proteins from aggregation, assist them in refolding or promote their proteolysis, thereby restoring cellular homeostasis (Wang et al., 2004; He et al., 2018). By reducing H_2_O_2_ to water, APXs are key components of the ROS scavenging system that copes with oxidative stress (Pang et al., 2011; Li et al., 2012; Cao et al., 2017). Therefore, NO_2_-18:3 is capable of motivating two general defense systems, which might be a conserved mechanism adopted by both plants and animals.

### Regulators of Stress Signaling

Apart from being components of signaling lipids such as PA [see reviews (Zhao, 2015; Hou et al., 2016)], C18 UFAs *per se* are regulators of stress response. In fact, as aforecited, they can activate PLDδ to produce PA (Wang and Wang, 2001). The involvement of 18:1 in plant immunity against pathogen infection has long been disclosed by the *suppressor of SA insensitive 2* (*ssi2*) mutant of *Arabidopsis*, which is deficient in SSI2/FAB2, the major SAD isoform (Kachroo et al., 2001). The mutant has higher resistance to the biotrophic fungus *Peronospora parasitica*, owing to the accumulation of salicylic acid (SA), another stress hormone, and the constitutive activation of its downstream pathogenesis-related (*PR*) genes. However, a concurrent phenotype is higher susceptibility to the necrotrophic fungus *Botrytis cinerea*, resulting from the impairment in some branches of JA response, e.g., defensin (*PDF1.2*) induction. Of note, this cannot be rescued by exogenous JA and it is the reduced level of chloroplast 18:1 that accounts for the alterations in the two hormonal signaling pathways [see review (Lim et al., 2017)].

Later, it was elucidated that 18:1 regulates defense signaling *via* suppressing NO production (Mandal et al., 2012). In plants, besides nitrate reductase (NR), NITRIC OXIDE ASSOCIATED 1 (NOA1), a protein bearing GTPase activity, is involved in NO biosynthesis, though the mechanism remains unknown. In chloroplast nucleoids, 18:1 physically interacts with NOA1 and promotes its degradation in a protease-dependent manner. Decreased 18:1 content thus leads to increased NOA1 level. Meanwhile, the expression of NIA1 and NIA2, two NRs, is also up-regulated. As a result, NO accumulates to launch its downstream signaling, thereby reprogramming the immune response, with SA and JA pathways being affected. Notably, *ssi2* phenotypes can be fully restored by disrupting NOA1 together with either NR.

However, 18:3 may regulate defense signaling *via* enhancing ROS production (Yaeno et al., 2004). The hypersensitive response (HR) of plants to prevent pathogen spread is characterized by oxidative burst and programmed cell death (PCD), with NOX being the key enzyme. *In vitro* assay demonstrated that 18:3 could effectively enhance NOX activity, suggesting its regulatory role in HR. Indeed, the *fad7fad8* double mutant of *Arabidopsis*, which is defective in chloroplast 18:3 synthesis, showed reductions in ROS level, PCD progress, and disease resistance following inoculation with *P. syringae* DC3000. Of note, the regulatory roles of 18:1 and 18:3 are not confined to biotic stresses, since NO and ROS are also potent organizers in abiotic stress response.

## Genetic Engineering of Fatty Acid Synthetic Genes for Improving Stress Tolerance

There are a growing number of cases succeeding in improving the ability of plants to deal with various abiotic stresses *via* genetically manipulating FA synthetic enzymes, in particular ω-3 FADs and plastidial GPAT (examples listed in **Table 2**). It should be highlighted that genes from extremophytes are good candidates for transgenesis, as exemplified by *FAD3* from *Chorispora bungeana*, a perennial crucifer inhabiting periglacial regions (Shi et al., 2018), and *GPAT* from *Ammopiptanthus mongolicus*, an evergreen broadleaf shrub inhabiting desert (Xue et al., 2019). Both of them conferred multistress tolerance on transgenic plants. Nevertheless, gene silencing of *FAD7* in tobacco (Murakami et al., 2000) or *GPAT* in tomato (Sui et al., 2007) enabled plants to endure high temperatures. Therefore, in order not to compromise heat tolerance, using synthetic stress-inducible promoters (Hou et al., 2012) to drive gene expression could be a method of choice.

Plastidial GPAT has attracted much interest in genetic engineering in that its substrate selectivity determines the unsaturation degree of chloroplast PG. GPAT from chilling-resistant spinach (*Spinacia oleracea*), but not chilling-sensitive squash (*Cucurbita moschata*), has a pronounced preference for 18:1-ACP, which matters because the second acylation almost exclusively utilizes 16:0-ACP [see review (He et al., 2020)]. An interesting phenomenon is that the chilling behavior of tobacco (intermediate) was shaped by GPATs from squash (sensitive) and *Arabidopsis* (resistant), respectively (Murata et al., 1992), whereas the salt susceptibility of *Arabidopsis* (glycophyte) was relieved by GPAT from *Suaeda salsa* (euhalophyte) (Sui et al., 2017). In fact, *S. salsa* also exhibited chilling tolerance under saline situations (Cheng et al., 2014).

To favor the anabolic flow toward C18 UFAs, other enzymes can be manipulated together with FADs. Candidates include acetyl-CoA carboxylase (ACC), 3-ketoacyl-ACP synthase II (KASII), and acyl-ACP thioesterase B (FATB) (**Figure 2**). ACC catalyzes the first step of FA synthesis and the plastid-targeting expression of *Arabidopsis ACC1* in *Brassica napus* increased 18:1 content (Roesler et al., 1997). KASII elongates 16:0-ACP to 18:0-ACP and its transactivation in *B. napus* increased total C18 FA content (Gupta et al., 2012). FATB releases 16:0 and 18:0 from ACP and its knockdown by an artificial microRNA in *Camelina sativa* increased 18:1 content (Ozseyhan et al., 2018). In addition, as essential cofactor for FA synthesis in plastids, ACP can also affect FA composition and stress tolerance. For example, introducing *ACP1* from peanut into tobacco resulted in higher levels of 18:2 and 18:3 coupled with lower susceptibility to cold (Tang et al., 2012).

Last but not least, it was newly reported that strong co-suppression may pose an obstacle to direct *FAD2* overexpression (Du et al., 2019). This can be overcome by the mutation of *RDR6* in *Arabidopsis*; however, the expense of impaired post-transcriptional gene silencing will be increased viral sensitivity and disturbed gene regulation. But for the transgenerational instability due to co-suppression (Du et al., 2019), the undesired effect can instead be taken advantage of to achieve high 18:1 content that is good for oil stability and human health. In fact, to this end, *FAD2* has become a “hotspot” for targeted disruption by using the powerful genome editing tool CRISPR/Cas9 (He et al., 2020). Nevertheless, it should be noted that, in *Arabidopsis*, FAD2 is required for establishing a proper ratio of 18:2 to 18:1 that allows the ER membrane to tolerate ER stress (Nguyen et al., 2019).

## Conclusion and Perspectives

In stress response, C18 UFAs are deeply involved and strikingly versatile. A notable feature is that the chemical nature of double bond appears to render them redox sensors *in planta*. Remarkably, oxylipins, the inevitable products from ROS attack, are also exploited in a smart way like ROS; that is, they are actively produced by specific enzymes as signaling molecules or biotoxins to retort the elicitors. It should be pinpointed that C18 UFAs are also “Janus” molecules that each has a positive face as general defender against biotic and abiotic stresses in concomitance with a negative face as potential inducer of oxidative stress. Moreover, there are still some aspects of FA/acyl-lipid metabolism remain to be elucidated, particularly the trafficking of the intermediates between different cellular compartments and the functions of a large number of lipases that are tagged hitherto as predicted.

Land plants are routinely confronted with an unpredictable combination of stresses in the field, especially under the context of climate change, soil salinization, and environmental pollution. It is thus getting imperative to equip them with multistress tolerance (He et al., 2018). The ability of C18 UFAs to combat a broad spectrum of stresses renders their synthetic genes good candidates for genetic engineering, as has been proven by the foregoing successful cases. Of note, C18 UFAs draw much interest not only for anti-stress roles, but for wholesome properties and industrial applications (He et al., 2020). Therefore, manipulating FA composition and increasing oil yield of crops are also prompted by the ever-increasing demand for high-quality edible oils and staple oleochemicals.

## Author Contributions

All authors listed have made a substantial, direct and intellectual contribution to the work, and approved it for publication.

## Funding

This work was supported by grants from Key R&D project of Shandong Province (2019GSF107020).

## Conflict of Interest

The authors declare that the research was conducted in the absence of any commercial or financial relationships that could be construed as a potential conflict of interest.
